# 

**Published:** 1860-08

**Authors:** 


### CONTENTS.

### ORIGINAL COMMUNICATIONS.

PAGE.

ARTICLE I.—Typhoid Fever; By V. H. TALIAFERRO, M. D., Columbus, Georgia, .......... 705

ARTICLE II.—Sabbath Clinical Benevolence; By ALEX. MEANS, M. D., Oxford, Georgia, .......... 716

### SELECTIONS.

American Medical Association. Thirteenth Annual Meeting, held at New Haven, June 5th, 6th and 7th, 1860, .......... 727

### EDITORIAL AND MISCELLANEOUS.

American Medical Association, .......... 752

Convention of Medical Teachers, .......... 753

Close of the Volume, .......... 754

New Orleans School of Medicine, .......... 754

Medical Education, .......... 755

Dr. Hay's Arctic Expedition.—The Views of its Commander, .......... 756

The Vaccination of Indians, .......... 759

Doctors and Physic, .......... 759

Index to Volume Fifth, .......... 767

Quackery Rampant, .......... 767

Meteorological Observations, .......... 768

